# Characterization of Wood-Plastic Composites Made with Different Lignocellulosic Materials that Vary in Their Morphology, Chemical Composition and Thermal Stability

**DOI:** 10.3390/polym9120726

**Published:** 2017-12-17

**Authors:** Ke-Chang Hung, Heng Yeh, Teng-Chun Yang, Tung-Lin Wu, Jin-Wei Xu, Jyh-Horng Wu

**Affiliations:** Department of Forestry, National Chung Hsing University, Taichung 402, Taiwan; d9833004@mail.nchu.edu.tw (K.-C.H.); guitarsoulinforest@gmail.com (H.Y.); tcyang.04@nchu.edu.tw (T.-C.Y.); tonywuwu22@gmail.com (T.-L.W.); ecsgunro@gmail.com (J.-W.X.)

**Keywords:** wood-plastic composites, lignocellulosic fibers, physico-mechanical properties, thermal decomposition kinetic, apparent activation energy

## Abstract

In this study, four kinds of lignocellulosic fibers (LFs), namely, those from Chinese fir (*Cunninghamia lanceolata*), Taiwan red pine (*Pinus taiwanensis*), India-charcoal trema (*Trema orientalis*) and makino bamboo (*Phyllostachys makinoi*), were selected as reinforcements and incorporated into high-density polyethylene (HDPE) to manufacture wood-plastic composites (WPCs) by a flat platen pressing process. In addition to comparing the differences in the physico-mechanical properties of these composites, their chemical compositions were evaluated and their thermal decomposition kinetics were analyzed to investigate the effects of the lignocellulosic species on the properties of the WPCs. The results showed that the WPC made with Chinese fir displayed a typical M-shaped vertical density profile due to the high aspect ratio of its LFs, while a flat vertical density profile was observed for the WPCs made with other LFs. Thus, the WPC made with Chinese fir exhibited higher flexural properties and lower internal bond strength (IB) than other WPCs. In addition, the Taiwan red pine contained the lowest holocellulose content and the highest extractives and α-cellulose contents, which gave the resulting WPC lower water absorption and flexural properties. On the other hand, consistent with the flexural properties, the results of thermal decomposition kinetic analysis showed that the activation energy of the LFs at 10% of the conversion rate increased in the order of Taiwan red pine (146–161 kJ/mol), makino bamboo (158–175 kJ/mol), India-charcoal trema (185–194 kJ/mol) and Chinese fir (194–202 kJ/mol). These results indicate that the morphology, chemical composition and thermal stability of the LFs can have a substantial impact on the physico-mechanical properties of the resulting WPCs.

## 1. Introduction

Wood is an anisotropic material and is thus susceptible to deformation, warping and cracking due to dimensional instability from changes in ambient humidity. Meanwhile, wood is susceptible to degradation caused by weathering and biological factors, which restricts wood to specific exterior applications [1]. However, mixing wood fiber with thermoplastic polymers to manufacture wood-plastic composites (WPCs) could mitigate these drawbacks. In addition, incorporation of wood fibers into plastics not only improves the flexural and tensile properties of the resulting composites relative to pure plastics [2] but also gives the composites many advantages, such as low equipment abrasiveness, relatively low cost, low maintenance requirements and environmental friendliness [3,4]. Therefore, WPCs are of great interest for residential products as well as in automotive applications and the construction industry. In fact, these WPCs can serve as alternatives to solid wood for various fixtures, such as window frames, fencing, roofing, decking and siding [5].

It is well known that the composition of the major chemical species present, i.e., cellulose, hemicellulose and lignin, significantly affect the physical and mechanical properties of lignocellulosic materials. The chemical constituents of different lignocellulosic materials have distinct differences. The performance of the WPCs depends on composite’s microstructure, void content, wood-matrix stress transfer efficiency, particle morphology and the chemical composition of the lignocellulose [6,7,8,9,10]. However, little information is available on the effect of various lignocellulosic fibers (LFs) on the properties of WPC. Accordingly, an objective of the current study was to investigate the effects of the morphology and chemical composition of the lignocellulosic materials on the physico-mechanical properties of the WPCs. Three common fast-growing plantation species and one common economical and popular bamboo species in Taiwan, Chinese fir (*Cunninghamia lanceolata*), Taiwan red pine (*Pinus taiwanensis*), India-charcoal trema (*Trema orientalis*) and makino bamboo (*Phyllostachys makinoi*), were used as reinforcements or fillers to prepare WPCs in this study. LFs are subjected to thermal degradation during composite processing [11,12]. Therefore, this study is of practical significance to understand the thermal decomposition behavior of LFs, and these data will aid in the design of composite processes and help estimate the influence of the thermal decomposition of natural fibers on the properties of composite [13]. The thermal decomposition kinetics of these lignocellulosic materials were evaluated using thermogravimetric analysis (TGA) by various isoconversional methods.

## 2. Experimental

### 2.1. Materials

Makino bamboo was provided by a local bamboo-processing factory. India-charcoal trema, Taiwan red pine and Chinese fir samples were obtained from the experimental forest of National Chung Hsing University in Nantou County, Taiwan. Lignocellulosic fibers (LFs) were prepared by hammer milling and sieving, and fibers between 16 and 24 mesh (*ϕ*1.00–0.71 mm) were investigated. High-density polyethylene (HDPE, LH901) with a melt index of 0.95 g/10 min and a density of 953 kg/m^3^ was purchased from USI Co. (Kaohsiung, Taiwan). The chemicals and solvents used in this experiment were purchased from Sigma-Aldrich Chemical Co. (St. Louis, MO, USA).

### 2.2. Composite Processing

To manufacture the WPCs, the flat-platen pressing process was applied as reported in our previous papers [14,15,16,17]. The weight ratio of oven-dried LF to HDPE powder was 60:40. The expected density of the WPCs was 900 kg/m^3^. The expected dimensions of the WPC samples were 200 mm × 300 mm with a thickness of 12 mm. All the WPCs were produced in a two-step pressing process involving (1) hot pressing (2.5 MPa) at 180 °C for 8 min and then (2) cold pressing until the temperature of the WPC decreased to 25 °C (ca. 12 min).

### 2.3. Morphological Characteristics of the Lignocellulosic Materials

The morphological characteristics of the LFs were analyzed by a stereo microscope (M500, MOTIC, Kowloon, China) equipped with a digital CCD camera (STC-TC83USB-A, Sentech, Tokyo, Japan) and image analysis software (MultiCam EZ 2007 v2, Shengtek, Taoyuan, Taiwan). The aspect ratios (length/width) of the LFs were calculated according to their morphological characteristics. At least forty specimens of each kind of LF were analyzed.

### 2.4. Chemical Composition Analysis of Lignocellulosic Materials

The contents of extractives, holocellulose, α-cellulose, and Klason lignin for various lignocellulosic materials were determined according to ASTM test methods D1107-96, D1104-56, D1106-96 and D1103-60, respectively. The amounts are expressed as a percentage of the initial oven-dry weight.

### 2.5. Determination of Composite Properties

To determine the properties of the composites, several determinations, including density, water absorption, thickness swelling, flexural properties, internal bond strength (IB) and wood screw holding strength (WS) were made according to ASTM D1037-06a and ASTM D790-07. The IB and WS were determined on samples with dimensions of 50 mm × 50 mm × 12 mm at a tensile speed of 2 mm/min. Specimens 230 mm × 50 mm × 12 mm were used to evaluate flexural strength (modulus of rupture, MOR) and modulus of elasticity (MOE) by the three-point static bending test with a loading speed of 10 mm/min and span of 180 mm. All the samples were conditioned at 20 °C and 65% relative humidity for two weeks prior to testing, and at least five specimens of each blend were tested. The flexural properties are calculated by the following equations:

(1) $$\mathrm{MOR}\;\left(\mathrm{MPa}\right)=\frac{3P_{\max}L}{2bh^{2}}$$

(2) $$\mathrm{MOE}\;\left(\mathrm{MPa}\right)=\frac{FL^{3}}{4\delta bh^{3}}$$

where *P*_max_ is the maximum load (N), *L* is the span of the specimen (mm), *b* is the width of the specimen (mm), *h* is the thickness of the specimen (mm) and *δ* is the mid-span deflection (mm) of the specimen corresponding to load *F* (N) at a point in the straight-line portion of the load-deflection curve.

### 2.6. Evaluation of the Vertical Density Profile

The vertical density profiles of specimens were analyzed by a QTRS-01X X-ray density profiler (Quintek Measurement Systems, Knoxville, TN, USA). Specimens with a size of 50 mm × 25 mm × 12 mm were scanned in the thickness direction, and the measurements were taken at 0.04 mm intervals. Ten specimens of each blend were tested.

### 2.7. Thermal Decomposition Kinetics Analysis

A Pyris 1 TGA (PerkinElmer, Shelton, CT, USA) was used to study the thermal properties of various lignocellulosic materials. Measurements on 3 mg samples were carried out in a nitrogen atmosphere (20 mL/min) from 50 to 600 °C. The heating rates were set at 10, 20, 30, 40 and 50 °C/min. The data obtained were used to calculate the kinetics parameters by model-free isoconversional methods. The conversion rate, *α*, is defined as follows:

(3) $$\alpha =\frac{W_{0}-W_{t}}{W_{0}-W_{f}}$$

where *W*_0_ is the initial weight of the sample, *W*_f_ is the final residual weight and *W*_t_ is the weight of the oxidized or pyrolyzed sample at time *t*. The common isoconversional methods used in this study include Friedman (Equation (4)), Flynn-Wall-Ozawa (F-W-O) (Equation (5)) and modified Coats-Redfern (modified C-R) (Equation (6)). The methods are represented by the following equations:

(4) $$\ln\frac{d\alpha }{dt}=\ln\left[Af\left(\alpha \right)\right]-\frac{E_{a}}{RT}$$

(5) $$\log\beta =\log\frac{AE_{a}}{Rg\left(\alpha \right)}-2.315-0.4567\frac{E_{a}}{RT}$$

(6) $$\ln\frac{\beta }{T^{2}\left(1-\frac{2RT}{E_{a}}\right)}=\ln\frac{-AR}{E_{a}\ln\left(1-\alpha \right)}-\frac{E_{a}}{RT}$$

where *α* is the conversion rate, *A* is the pre-exponential factor (min^−1^), *f*(*α*) is the reaction model, *E*_a_ is the apparent activation energy (kJ/mol), *R* is the gas constant (8.314 J/K/mol), *T* is the absolute temperature (K), *β* is the heating rate and *g*(*α*) is a function of the conversion [13,18,19]. Therefore, for a given conversion fraction, linear relationships are observed by plotting ln(d*α*/d*t*), log*β* and ln(*β*/*T*^2^) versus 1/*T* at different heating rates, and then *E*_a_ is calculated from the slope of the straight line.

### 2.8. Analysis of Variance

All the results are expressed as the mean ± standard deviation (SD). The significance of the difference was calculated by Scheffe’s test; *p* value < 0.05 was considered significant.

## 3. Results and Discussion

### 3.1. Morphological Characteristics and Chemical Compositions of Different LFs

**Figure 1:**
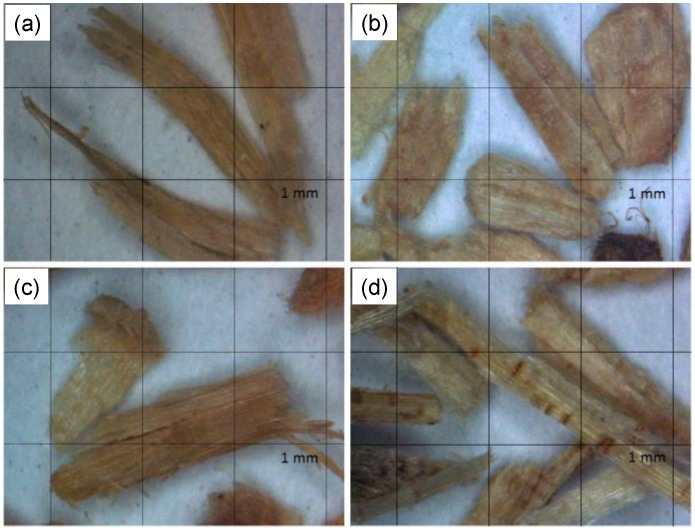
Micrographs of different lignocellulosic fibers: (**a**) makino bamboo; (**b**) India-charcoal trema; (**c**) Taiwan red pine; and (**d**) Chinese fir.

The images of the morphologies of various LFs are shown in Figure 1. It can be noted that the morphological characteristics of the four LFs are different, which can be attributed to the differences in structural, physical and mechanical properties of the lignocellulosic materials. The fiber morphology and chemical composition of the various lignocellulosic materials are listed in Table 1. The lengths of the LF of makino bamboo, India-charcoal trema, Taiwan red pine and Chinese fir were 3.1, 2.0, 2.5 and 3.2 mm, respectively, while the aspect ratios were 4.4, 2.9, 3.3 and 6.3, respectively. Accordingly, the LFs of makino bamboo and Chinese fir appear longer and more slender than those of India-charcoal trema and Taiwan red pine. These undesirable differences are explained by the fact that the different lignocellulosic materials responded differently to the milling and sieving processes. The higher strength of the lignocellulosic materials in their axial direction is likely to produce long fibers and high aspect ratios. This fibrous behavior is usually promoted by high cellulose contents and low microfibril angles [6]. It was also noted that LFs oriented lengthwise can pass through the sieve aperture. These results show that different species of LFs can pass through the same sieve size, but their morphologies can be different.

### 3.2. Effect of Different LFs on Physical Properties of the WPCs

**Figure 2:**
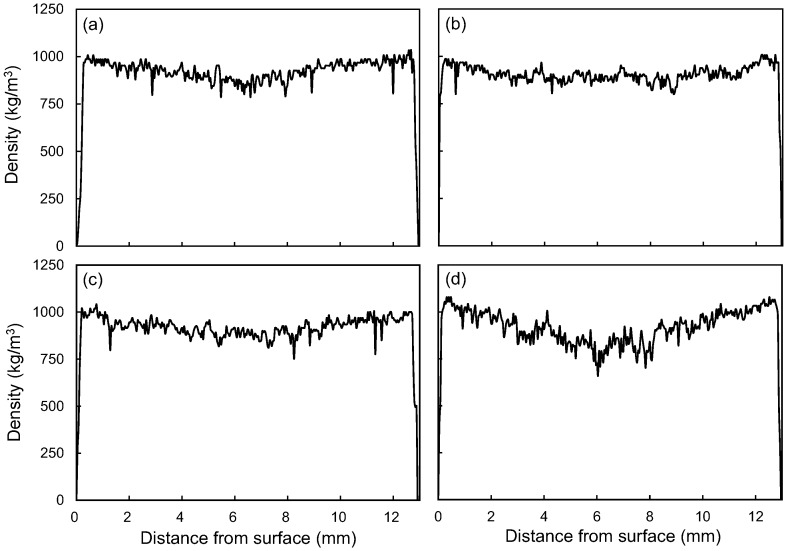
The vertical density profiles of WPCs made with makino bamboo (**a**); India-charcoal trema (**b**); Taiwan red pine (**c**); and Chinese fir (**d**).

The various physical properties of the WPC with 60 wt % of LFs are shown in Table 2. In general, the density and moisture content can affect the mechanical properties of the polymer composites. The densities and moisture contents of all composites are approximately 900–915 kg/m^3^ and 4.3–4.5%, respectively; no significant differences were noted among these results. In addition, after 24 h of immersion in water, the WPC made with Taiwan red pine exhibited the lowest water absorption (7.3%) and thickness swelling (3.6%). This phenomenon may be affected by the high extractive content of Taiwan red pine (11.7%), which is rich in hydrophobic abietic acids [20]. On the other hand, the Taiwan red pine also contained the least amount of holocellulose (61.0%) (Table 1), thus the Taiwan red pine had fewer hydrophilic hydroxyl groups than other lignocellulosic materials. Interestingly, although the holocellulose content of Chinese fir (66.4%) was lower than those of makino bamboo (71.0%) and India-charcoal trema (73.7%), the WPC made with Chinese fir had the highest water absorption (15.4%) and thickness swelling (6.8%). The water absorptions of WPCs are mainly due to the gaps and flaws within the composites as well as the hydrophilicity of the natural fibers [21]. The vertical density profile of the WPCs may be the reason for this occurrence. As shown in Figure 2, the vertical density profile of the WPC made with Chinese fir presents a typical M-shaped curve, indicating the composite has a low core density. Hence, the WPC made with Chinese fir has more gaps and flaws in the core layer than the other composites do. Additionally, the highest aspect ratio of the Chinese fir fibers could be another explanation for the water absorption and thickness swelling. As suggested by Migneault et al. [22], the high aspect ratio of the fibers increases the water absorption and thickness swelling of the resulting composites.

### 3.3. Effect of Different LFs on Mechanical Properties of the WPC

**Figure 3:**
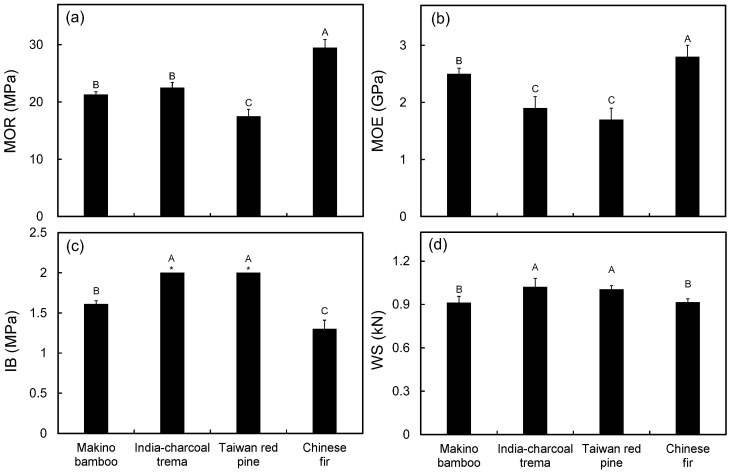
The MOR (**a**); MOE (**b**); internal bond strength (IB) (**c**); and wood screw holding strength (WS) (**d**) of WPCs made with different lignocellulosic fibers. *: IB over the test limitation (2 MPa). Values are the mean ± SD (*n* = 5). Different letters indicate significant differences among groups (*p* < 0.05).

The effect of various lignocellulosic materials on mechanical properties, such as MOR, MOE, IB and WS, are given in Figure 3. The highest MOR was found in the WPC made with Chinese fir (29.5 MPa), followed by the WPCs made with India-charcoal trema (22.5 MPa), makino bamboo (21.3 MPa) and Taiwan red pine (17.5 MPa). This result seems to reveal that the LFs with the highest aspect ratios generate composites with superior MORs, and this is in agreement with previous reports [8,23,24]. However, the MOR of a WPC is not only influenced by the aspect ratio of the LFs but also influenced by the vertical density profile of the composite and the stiffness of the reinforcements. The stiffness of LFs depends on their crystallinity; thus, the stiffness is associated with the α-cellulose content [10]. The Taiwan red pine had the lowest α-cellulose content, resulting in its WPC having the lowest MOR. In contrast, the Chinese fir has the highest α-cellulose and lignin contents. It is well known that lignin acts as a matrix that holds cellulose fibers together. Diffusion of lignin into the fiber walls increases stiffness and allows stress transfer between the matrix and the cellulose fibers [25]. Therefore, the WPC made with Chinese fir exhibited the highest MOR due to the specific lignocellulosic characteristics and the higher density in both face layers of the composite. Moreover, the MOE values showed a trend similar to what was seen for the MOR values (Figure 3b); the MOE values decrease in the following order: Chinese fir (2.8 GPa), makino bamboo (2.5 GPa), India-charcoal trema (1.9 GPa) and Taiwan red pine (1.7 GPa).

Furthermore, IB is one of the important mechanical properties because it indicates the interfacial adhesion of the composites. Figure 3c shows that the IB of the various WPCs made with different LFs. Accordingly, the IBs of WPCs made with India-charcoal trema and Taiwan red pine are greater than those of WPCs made with makino bamboo and Chinese fir. This trend may be related to the vertical density profile of the WPCs since the composites with higher core densities generally exhibit higher IBs [26]. As shown in Figure 2, more uniform density profiles, i.e., equal surface and core density, were noted in the WPCs made with India-charcoal trema (Figure 2b) and Taiwan red pine (Figure 2c). In contrast, M-shaped vertical density profiles were observed in the WPCs made with makino bamboo and Chinese fir, especially in the latter one. Accordingly, the WPCs made with India-charcoal trema and Taiwan red pine displayed higher core densities, leading to higher IBs. A similar tendency was also observed for the WS of the WPCs, and the strengths ranged from 913 to 1022 N (Figure 3d).

### 3.4. Thermal Decomposition Kinetics of Different LFs

**Figure 4:**
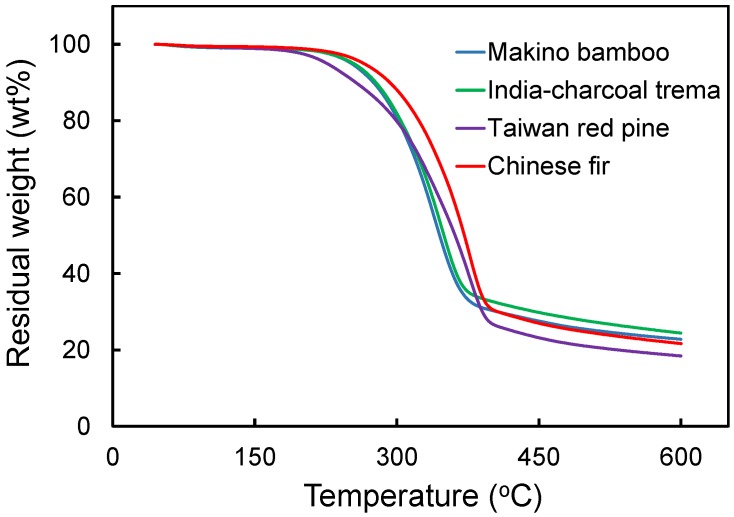
Thermogravimetric analysis of various lignocellulosic materials at a heating rate of 20 °C/min.

Lignocellulosic materials were subjected to thermal degradation during composite processing, which can affect the properties of the resulting composites [11,13]. Therefore, TGA was used to investigate the thermal decomposition behavior of the four kinds of lignocellulosic materials. Figure 4 shows the TGA curves of the lignocellulosic materials at a heating rate of 20 °C/min. Among the materials, the Taiwan red pine displayed an obvious weight loss above 220 °C, while the initial weight loss temperature of Chinese fir was approximately 60 °C higher, indicating it is more thermally stable. This result revealed that the thermal decomposition mechanisms of lignocellulosic materials are significantly different.

**Figure 5:**
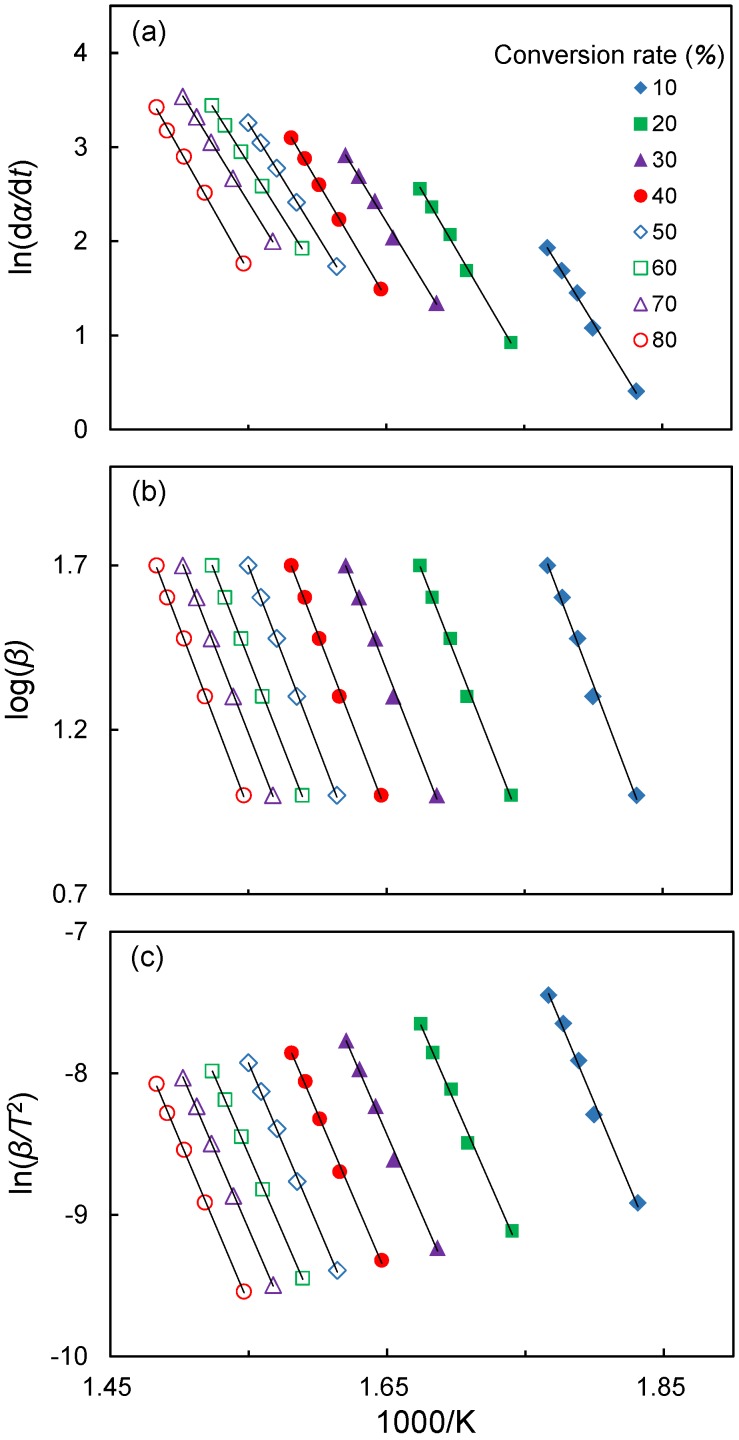
Typical isoconversional plots using the Friedman method (**a**); Flynn-Wall-Ozawa method (**b**); and Modified Coats-Redfern method (**c**) for Chinese fir.

**Figure 6:**
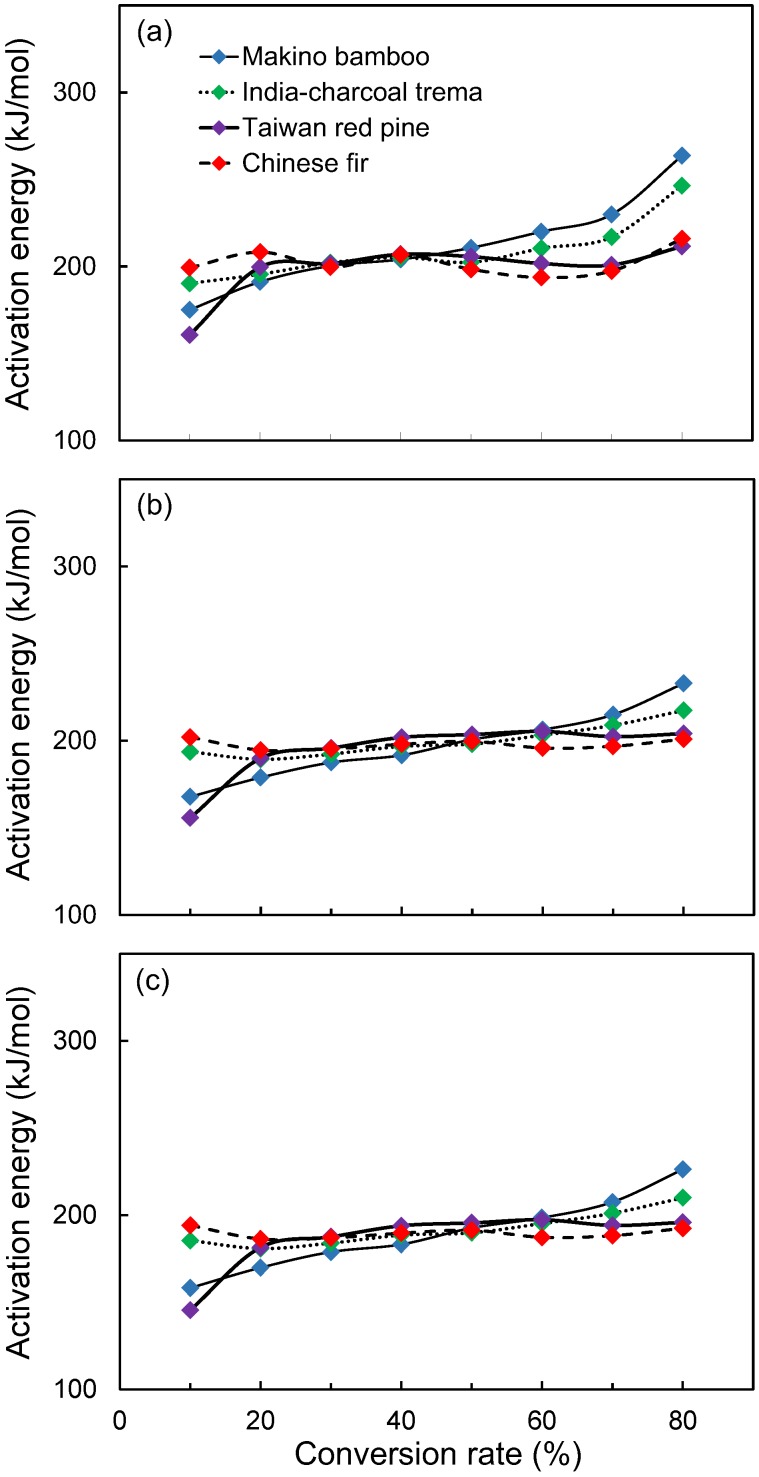
A comparison of apparent activation energies as a function of decomposition conversion rate (*α*) for various lignocellulosic fibers calculated by the Friedman method (**a**); Flynn-Wall-Ozawa method (**b**); and modified Coast-Redfern method (**c**).

To analyze the thermal decomposition mechanisms in depth, the Friedman, F-W-O and modified C-R methods were used. As an example, the Friedman, F-W-O and modified C-R plots for Chinese fir are shown in Figure 5. It is remarkable that the fitted lines are highly linear; thus, these methods are suitable to evaluate the activation energy of thermal decomposition at different conversion rates for the LFs. The plots of other lignocellulosic materials are similar to those presented in Figure 5 (not shown). The plots of activation energy as a function of conversion rate (in the conversion range of 10–80%) for all lignocellulosic materials based on the three methods were also prepared. As shown in Figure 6, the *E*_a_ values of various lignocellulosic materials are significantly different at lower conversions (*α* < 20%) and higher conversions (*α* > 60%). However, the temperature used to prepare the composite does not exceed the temperature at 10% conversion. Accordingly, the *E*_a_ at 10% conversion was used to evaluate the thermal stability of the lignocellulosic materials under the conventional processing temperatures for composites. The correlation coefficient (*R*^2^) and the *E*_a_ at 10% conversion calculated from the abovementioned methods are listed in Table 3. As shown in Table 3, the *E*_a_ at 10% conversion of various lignocellulosic materials increase in order of Taiwan red pine (146–161 kJ/mol), makino bamboo (158–175 kJ/mol), India-charcoal trema (185–194 kJ/mol) and Chinese fir (194–202 kJ/mol). The *E*_a_ is defined as the minimum energy that must be overcome to start a chemical reaction [27]. As the *E*_a_ value increases, the minimum energy required to start thermal decomposition also increases. Accordingly, this result indicates that the Chinese fir was most thermally stable, while the Taiwan red pine was least stable.

## 4. Conclusions

After milling and sieving, the LFs of different lignocellulosic materials showed different morphologies. When a WPC is made with Chinese fir, which has fibers with a larger aspect ratio (length/width), its vertical density profile is M-shaped, which means the composite has better MOR and MOE values, but it also has higher water absorption and thickness swelling after 24 h of soaking. In addition, the contents of extractives and holocellulose within the LFs also affect the water absorption and thickness-swelling properties of the composites. The WPC made with Taiwan red pine, which contained the highest amount of extractives and the lowest amount of holocellulose, exhibited the lowest water absorption after 24 h of soaking. Moreover, among all the LFs used in this study, the highest activation energy of thermal decomposition at the 10% of conversion rate was observed for Chinese fir and the Taiwan red pine was the lowest. Accordingly, the performance of a WPC is affected by the lignocellulosic species. These results could be used to guide the preparation of controllable and optimized composites for specific applications, such as mechanical- or moisture-resistant decking materials.

## Figures and Tables

**Table 1:** Morphology and chemical composition of different lignocellulosic fibers.

Characteristics of Lignocellulosic Fiber	Lignocellulosic Materials			
	Makino Bamboo	India-Charcoal Trema	Taiwan Red Pine	Chinese Fir
Morphology				
Length (mm)	3.1 ± 0.9 ^A^	2.0 ± 0.4 ^C^	2.5 ± 0.7 ^B^	3.2 ± 0.8 ^A^
Width (mm)	0.8 ± 0.3 ^A^	0.7 ± 0.2 ^A^	0.8 ± 0.2 ^A^	0.6 ± 0.2 ^B^
Aspect ratio	4.4 ± 1.8 ^B^	2.9 ± 1.3 ^C^	3.3 ± 1.5 ^C^	6.3 ± 2.7 ^A^
Chemical composition				
Holocellulose (%)	71.0 ± 0.8 ^B^	73.7 ± 0.2 ^A^	61.0 ± 1.3 ^D^	66.4 ± 0.3 ^C^
α-Cellulose (%)	43.7 ± 0.4 ^A^	42.6 ± 0.9 ^A^	37.7 ± 1.1 ^B^	45.1 ± 1.0 ^A^
Lignin (%)	20.9 ± 3.8 ^B^	25.4 ± 3.1 ^B^	28.3 ± 0.9 ^B^	37.1 ± 1.5 ^A^
Extractives (%)	4.0 ± 0.2 ^B^	4.4 ± 0.2 ^B^	11.7 ± 0.3 ^A^	4.0 ± 0.1 ^B^

Values are mean ± SD (*n* = 5). Different letters within a row indicate significant difference at *p* < 0.05.

**Table 2:** The physical properties of WPCs made with different lignocellulosic fibers.

Lignocellulosic Materials	Density (kg/m^3^)	Moisture Content (%)	24 h Soaking	
			Water Absorption (%)	Thickness Swelling (%)
Makino bamboo	911 ± 17 ^A^	4.5 ± 0.2 ^A^	12.2 ± 1.5 ^B^	4.1 ± 0.3 ^B^
India-charcoal trema	904 ± 15 ^A^	4.3 ± 0.2 ^A^	9.0 ± 1.7 ^BC^	4.5 ± 0.3 ^B^
Taiwan red pine	914 ± 12 ^A^	4.4 ± 0.2 ^A^	7.3 ± 1.2 ^C^	3.6 ± 0.8 ^B^
Chinese fir	915 ± 21 ^A^	4.3 ± 0.1 ^A^	15.4 ± 2.1 ^A^	6.8 ± 0.8 ^A^

Values are mean ± SD (*n* = 5). ^A,B,C^ Different letters in superscript indicate significant difference among groups (*p* < 0.05).

**Table 3:** Apparent activation energy of different lignocellulosic fibers calculated by three isoconversional methods at the *α* = 10%.

Lignocellulosic Materials	Friedman		F-W-O		Modified C-R	
	*E*_a_ (kJ/mol)	*R*^2^	*E*_a_ (kJ/mol)	*R*^2^	*E*_a_ (kJ/mol)	*R*^2^
Makino bamboo	175	0.9890	168	0.9895	158	0.9870
India-charcoal trema	190	0.9984	194	0.9958	185	0.9949
Taiwan red pine	161	0.9941	156	0.9836	146	0.9794
Chinese fir	199	0.9955	202	0.9931	194	0.9917
